# Effects of short-term warm water immersion on cardiac baroreflex sensitivity in healthy men

**DOI:** 10.1186/s12576-020-00762-1

**Published:** 2020-07-09

**Authors:** Jun Sugawara, Tsubasa Tomoto

**Affiliations:** 1 Human Informatics and Interaction Research Institute, National Institute of Advanced Industrial Science and Technology, 1-1-1 Higashi, Tsukuba, Ibaraki 305-8566 Japan; 2 Institute for Exercise and Environmental Medicine, Texas Health Presbyterian Hospital Dallas, Dallas, TX USA; 3 Department of Neurology and Neurotherapeutics, University of Texas Southwestern Medical Center, Dallas, TX USA

**Keywords:** Warm water immersion, Blood pressure, Cardiac baroreflex, Arterial stiffness

## Abstract

Warm water immersion (WWI) causes dizziness presumably due to a substantial drop of blood pressure. The aim of this study was to elucidate the effects of short-term WWI on cardiac baroreflex sensitivity (BRS) and the contribution of arterial stiffness to the cardiac BRS. Twelve apparent healthy men (44 ± 12 years) performed the single stand-up test after 5-min sitting in the bathtub without (Control) and with 41 °C warm water at the heart level (WWI). Cardiac BRS gain was evaluated by R–R interval response to the standing-induced drop of systolic blood pressure. In addition, before and 10 min after the single stand-up test, carotid arterial β-stiffness index was evaluated in the supine rest. BRS gain was blunted (2.9 ± 1.6 vs. 1.8 ± 1.1 ms/mmHg, *P* = 0.005), whereas β-stiffness index was not changed significantly after WWI. BRS gain correlated with β-stiffness index before (*r* = − 0.626, *P* = 0.028) and after WWI (*r* = − 0.672, *P* = 0.015). ANCOVA revealed that these slopes of linear regression lines remained unchanged after WWI (*P* = 0.350). These results indicate that a short-term WWI acutely deteriorates cardiac BRS. Individuals with stiffer arteries are relatively more susceptible to WWI because of their poor baseline BRS, which might be one of the causes of bathing-related falling in elderly persons as well as frailty.

## Background

Passive heat therapy seems to be capable of inducing improvements in vascular health [4, 17]. Thus, it could be a promising approach which has potential to reduce cardiovascular disease risk, incidence of cardiovascular-related mortality, sudden cardiac death, and all-cause mortality [12]. However, bath-related fatal (e.g., sudden cardiac arrest) and non-fatal accidents (e.g., dizziness and disturbance of consciousness) have been reported frequently in Japan that has a traditional custom to daily warm water immersion (WWI) in the bathtub at the bathing. Regarding non-fatal accidents, it is well-known empirically that immediately after WWI feeling dizzy on standing up frequently occurs presumably due to a substantial drop of blood pressure which could be a risk for fall-down and syncope. Yet, the underlying mechanism of bathing-related accidents remains to be determined.

Orthostatic challenge results in transient reductions of cardiac output and mean arterial pressure (MAP), and hence concomitant decreases in cerebral perfusion pressure and blood flow because blood is displaced from the thorax to the lower body [20]. In this situation, maintaining adequate blood pressure via baroreflex is likely to be an important regulatory mechanism in tolerating orthostatic hypotension and avoiding syncope [20]. Importantly, standing-induced reductions in cerebral perfusion tend to be exacerbated in the heat due to the large redistribution of central blood volume to the cutaneous vascular bed for thermoregulation [5]. Thus, several studies have been performed to investigate the effect of passive heat stress on baroreflex control of blood pressure and inconsistent results about impact on cardiac baroreflex sensitivity (BRS) are reported. The maximal gain between arterial pressure and heart rate (HR) was not changed significantly after whole body heating via water-perfused suits [7, 27, 28], and blunted cardiac BRS gain could be confirmed by use of the relationship between arterial pressure and R–R interval (RRI) [7]. Transfer function analysis reveals that passive heat stress reduces dynamic regulation of cardiac BRS gain [8]. In addition, heat stress blunts the response time of carotid-cardiac and carotid-vasomotor baroreflexes [29]. Thus, prolonged heat stress and hypohydration seem to impair orthostatic tolerance [5]. However, the impact of short-term WWI which might not to induce hypohydration on cardiac BRS has never been clarified.

A more important issue is that previous results may not provide us adequate answer to aforementioned question of underlying mechanism of non-fatal accidents immediately after WWI because of no-real world experimental setting (e.g., water-perfused suits and carotid baroreceptor-selective stimulation via neck chamber) [7, 8, 27–29]. In the case of bathing, getting out the bathtub is associated with integrated stimulations of (1) the combined unloading of aortic and carotid arterial baroreceptors and (2) absence of hydraulic compression to dilated vessels in the lower extremities. Therefore, we evaluated cardiac BRS at the action of getting out from the bathtub, the practical situation, in the present study.

Generally, blood pressure is well-regulated instantly by sensing fluctuation of blood pressure via baroreceptors located at the aortic arch and carotid arteries [18, 19]. Since these are mechanoreceptors, arterial stiffness is associated with sensitivity of baroreceptors [18]. Indeed, a large cohort study revealed that the increased arterial stiffness could be attributed to impaired cardiac baroreflex sensitivity [15] and exacerbated postural hypotension [16]. However, influence of WWI on the relationship between arterial stiffness and BRS remains unclear.

The aims of this study were to elucidate the effects of short-term WWI on cardiac BRS in healthy men, and the contribution of arterial stiffness to the change in cardiac BRS with WWI. The hypotheses of this study were that a short-term warm water bathing deteriorates cardiac BRS, and that individual changes in arterial stiffness with WWI are attributed to the response of cardiac BRS to WWI.

## Methods

### Subjects

To determine the contribution of arterial stiffness to the change in cardiac BRS with WWI, we set inclusion criteria were men aged between 20 and 60 years who were not medicated individuals, having overt cardiovascular diseases as assessed by medical history, and current smokers. Twelve healthy men (27–57 y.o., mean age of 44 ± 11 y.o., height of 171.0 ± 4.6 cm, weight of 67.0 ± 10.8 kg, body mass index of 23.0 ± 3.9 kg/m^2^, body fat of 17.3 ± 7.8%, mean ± SD) were studied.

### Experimental protocol

**Fig. 1:**
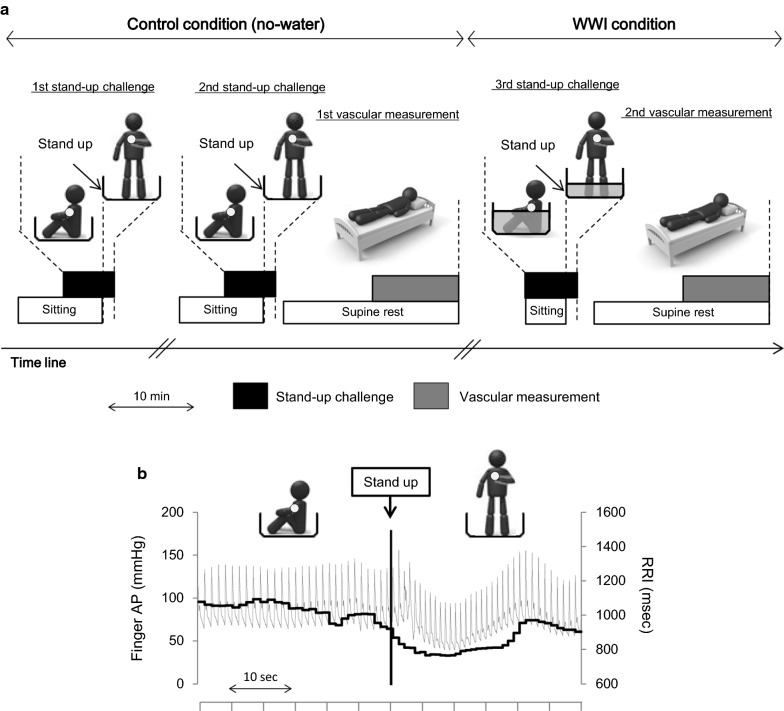
Experimental protocol (**a**) and typical response of finger arterial pressure waveform to the stand-up challenge (**b**). In **a**, white circles on human icons indicate the position of finger blood pressure cuff. The bathtub was empty in the control condition, whereas in the WWI condition warm water was kept in the bathtub at the heart level of each subject. “Stand-up challenge” (*black bars*) consisted of 5-min sitting and 1-min standing. In **b**, gray and black lines indicate finger arterial pressure (AP) and R–R interval (RRI), respectively

Subjects were instructed to abstain from alcohol intake and strenuous physical activity for at least 24 h prior to study. Furthermore, all measurements were performed after 3 h of fasting and an abstinence from caffeine. Upon arrival, subjects underwent height and body mass assessments. The following procedure (Fig. 1) was performed by wearing only swim shorts (no shirt). Each subject was instrumented for finger arterial pressure waveform measurement (via Finometer system) and received more than 5 min of quiet resting in the sitting posture on the floor of bathtub (no water) with arterial pressure recording. When blood pressure and HR (e.g., pulse rate) reached steady, each subject underwent the single stand-up test which consists 5-min sitting on the floor of the bathtub and 1-min standing in the bathtub. Each subject was instructed to keep quietly during sitting and standing. To confirm reproducibility of BRS evaluation, each subject repeated the single stand-up test twice (e.g., the first and second test) in the bathtub without water (control condition) with the 5-min interval. After the completion of 2 single stand-up tests, each subject was removed Finometer system and moved to the bed in a quiet, temperature-controlled room (24–26 °C). Each subject took 10-min supine rest which followed by the first vascular measurement. After the vascular measurement, subject returned to the bathroom and performed the third single stand-up test with re-instrumented Finometer system (5 min of sitting on the floor of bathtub with 41 °C warm water at the heart level, which was followed by 1 min of quiet standing in the bathtub: WWI condition). After this test, each subject wiped out his body quickly and took 10-min quiet supine rest, which was followed by the second vascular measurement.

### Measurements

#### Vascular measurements

HR and brachial blood pressure (BP) were measured with the cardiovascular screening device (VP-1000 plus; Omron Healthcare, Kyoto, Japan). Carotid arterial β-stiffness index, an index of arterial stiffness adjusted for distending pressure, was also measured as we previously reported [24]. In brief, the B-mode longitudinal ultrasound images of the left common carotid were recorded using an ultrasound device with a high-resolution (14 MHz) linear transducer (CX50xMATRIX; Philips Ultrasound, Bothell, WA) and were analyzed offline using automatic edge-detection software (Vascular Tool 5; Medical Imaging Applications, Coralville, IA). Carotid arterial pressure waveforms were obtained with arterial applanation tonometry incorporating an array of 15 micropiezoresistive transducers (VP-2000, Colin Medical, Komaki, Japan), and were calibrated by equating the carotid mean arterial and diastolic BP to the brachial mean arterial and diastolic BP [2]. Those were calibrated by equating the carotid mean arterial and diastolic BP to the brachial artery values. β-Stiffness index, an index of arterial compliance adjusted for distending pressure, was calculated using the following equation: ln(*P*_1_/*P*_0_)/[(*D*_1_ − *D*_0_)/*D*_0_], where *D*_1_ and *D*_0_ are the maximal and minimum diameters, and *P*_1_ and *P*_0_ are carotid arterial pressure at peak systole and end diastole.

#### Cardiac baroreflex sensitivity

Arterial pressure waveform was continuously recorded at the left middle finger by Finometer system (Finometer MIDI, Finapres Medical Systems, Amsterdam, The Netherlands) during the single stand-up test, and stored on a computer using a data acquisition system (PowerLab, AD Instrument) at the 1000 Hz of sampling rate. Throughout the test, subject was asked to keep the left hand at the heart level: on the side-table out of the bathtub during sitting and on the chest during standing. These data were analyzed offline by flexible data analysis software (LabChart, ADInstrument). Cardiac BRS gain was evaluated by the sequence method (e.g., the slope of linear regression line between standing-induced abrupt drop of systolic blood pressure (SBP) and corresponding reduction in RRI). RRI was estimated from the time interval between the systolic foot and the next systolic foot of arterial pressure waveforms. The slope of linear regression line was calculated from the continuous reduction of SBP and corresponding change in RRI (without time-lag) as long as possible (at least more than 3 beats). A correlation coefficient (*r*) > |0.85| was included in the analysis [28]. Hemodynamic variables during the single stand-up test were evaluated using a beat-to-beat hemodynamics analyzing software incorporated the validated stroke volume (SV) estimation (via Modelflow method [22, 25]). Finger systolic and diastolic BP, MAP, HR, SV, cardiac output (CO = HR × SV), and systemic vascular resistance (SVR = MAP/CO) were measured beat-to-beat manner throughout the single stand-up test (e.g., 5-min sitting, the stand-up motion, and 1-min standing). To determine the impact of WWI, averaged systemic hemodynamic variables were calculated from 3.5 to 4.5 min of sitting.

#### Body temperature

Tympanic membrane temperature was measured 30 s before the standing in both conditions by the infrared tympanic thermometer (Genius 2, GOVIDIEN, Mansfield, MA, USA).

### Statistical analyses

To evaluate reproducibility of BRS measurement, intra-class correlation coefficient (ICC) and 95% limits of agreement were calculated from two measurements of BRS gain at the control condition. Paired *t* test was applied for comparison between two conditions. Simple correlation analysis was performed to determine relations of interests. The slopes of linear regression line between β-stiffness index and BRS gain were compared by interaction between time and β-stiffness index on BRS gain by ANCOVA. All data are reported as mean ± SD. Statistical significance was set a priori at *P* values less than 0.05.

## Results

Table 1 summarizes systemic hemodynamics and tympanic temperature during sitting in the bathtub. HR and CO were significantly increased during WWI, whereas MAP, SV, SVR, and tympanic temperature did not change significantly.

**Table 1:** Tympanic temperature and systemic hemodynamics during sitting in the bathtub without (control) and with warm water (WWI)

Variables	Control	WWI	*P*-value
Tympanic temperature, °C	36.4 ± 0.3	36.4 ± 0.3	0.999
Heart rate, beats/min	62 ± 8	68 ± 8	0.017
Finger systolic BP, mmHg	107 ± 17	107 ± 17	0.961
Finger diastolic BP, mmHg	58 ± 14	62 ± 14	0.210
Finger MAP, mmHg	74 ± 15	77 ± 14	0.278
Stroke volume, mL	84.6 ± 17.2	83.5 ± 16.2	0.760
Cardiac output, L/min	5.2 ± 0.9	5.6 ± 1.2	0.022
Systemic VR, mmHg min/L	15.2 ± 6.3	14.6 ± 6.0	0.556

Data are mean and SD. Systemic hemodynamic variables were averaged values from 3.5 to 4.5 min of the sitting period

*BP* blood pressure, *MAP* mean arterial pressure, *VR* vascular resistance

**Fig. 2:**
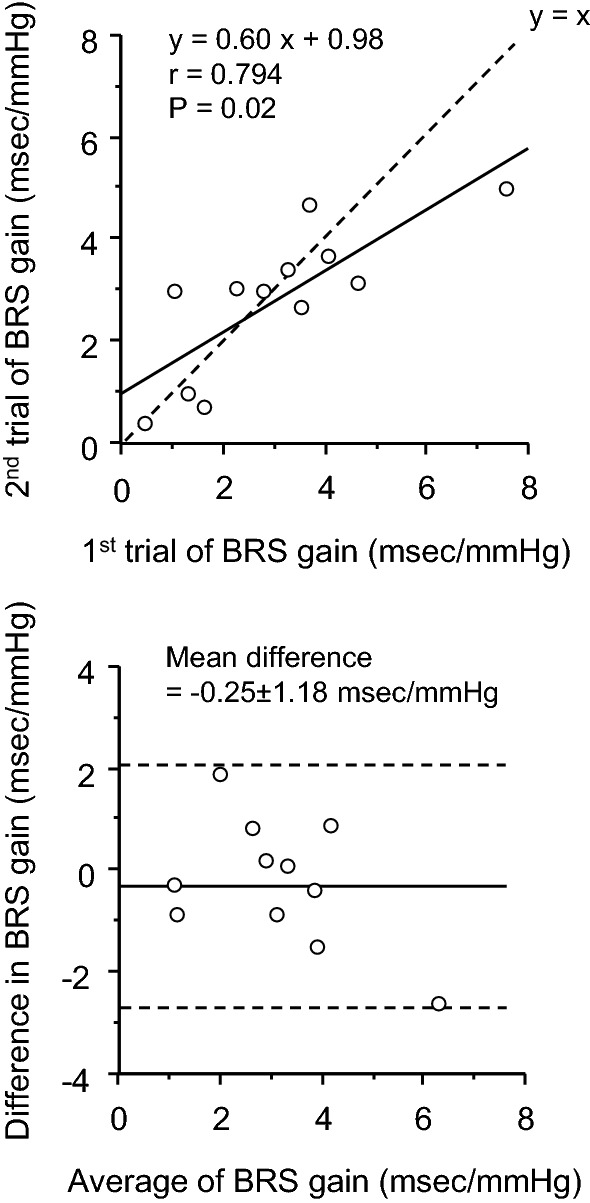
Reproducibility of cardiac baroreflex sensitivity (BRS) gain evaluated by the single stand-up test: scatter plot (**a**) and Brand and Altman’s plot (**b**)

Regarding reproducibility of BRS gain by the single stand-up challenge, ICC was 0.749 (*P* = 0.002, Fig. 2). Mean difference and 95% limits of agreement were − 0.25 ± 1.18 and from − 2.55 to 2.06 ms/mmHg, respectively. Thus, the averaged value of two bouts was calculated as BRS gain in the control condition.

**Fig. 3:**
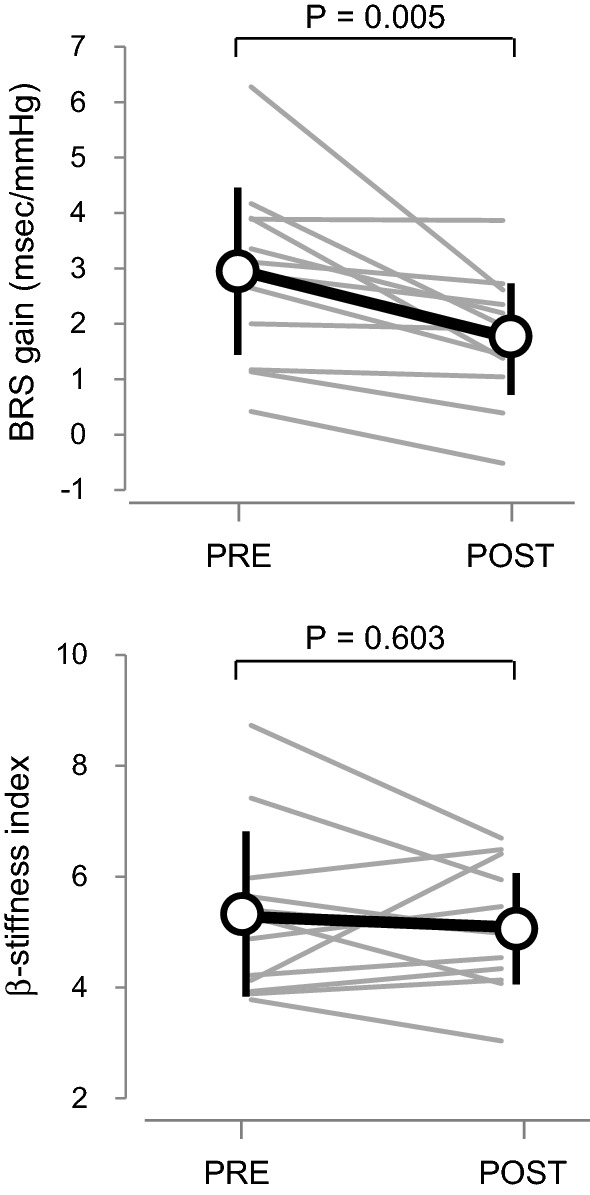
Cardiac baroreflex sensitivity (BRS) gain and carotid arterial β-stiffness index before (PRE) and after (POST) warm water immersion. Circles and error bars are mean and SD. Gray lines indicate individual changes

Figure 1 depicts typical response of MAP and RRI to a stand-up motion. There were no significant differences in either the baseline (sitting) finger artery MAP (from 73 ± 16 to 77 ± 16 mmHg, *P* = 0.322) or the standing-induced decreases in finger artery MAP (from − 33 ± 12 to − 33 ± 13 mmHg, *P* = 0.970) between two conditions (Table 2). The number of beats used for SBP-RRI slope calculation was significantly smaller in the WWI than the control condition (*P* = 0.007), where linear regression coefficients were equivalent between two conditions (*P* = 0.719). BRS gain was significantly blunted after the bathing (from 3.04 ± 1.93 to 1.78 ± 1.15 ms/mmHg, *P* = 0.014, Fig. 3).

**Table 2:** Hemodynamic response to the stand-up challenge without (control) and with warm water (WWI)

Variables	Control	WWI	*P*-value
Baseline HR, beat/min	62 ± 7	72 ± 9	0.003
Baseline MAP, mmHg	73 ± 16	77 ± 16	0.322
Nadir MAP, mmHg	43 ± 8	46 ± 12	0.364
Delta MAP, mmHg	− 33 ± 11	− 34 ± 14	0.700
Beat to nadir MAP, beats	13 ± 2	11 ± 4	0.235
Number of data plots, *n*	6.3 ± 1.5	4.8 ± 1.3	0.007
Correlation coefficient	0.964 ± 0.022	0.968 ± 0.027	0.719

Data are mean and SD. Baseline data were averaged values for 10 s immediately before the stand-up motion. *HR* heart rate, *MAP* finger artery mean arterial pressure. Number of data plot and correlation coefficient were obtained from the regression line calculation data between finger systolic blood pressure and R–R interval

Regarding vascular measurements, HR (from 56 ± 6 to 54 ± 6 beat/min, *P* = 0.025) and brachial diastolic BP (from 72 ± 12 to 69 ± 8 mmHg, *P* = 0.030) were significantly decreased in the 2nd measurement (e.g., 10 min after the end of WWI) than those in the 1st measurement (e.g., before WWI), whereas brachial systolic BP (from 114 ± 14 to 110 ± 11 mmHg, *P* = 0.126) and β-stiffness index (from 5.3 ± 1.5 to 5.1 ± 1.1, *P* = 0.603, Fig. 3) did not change significantly.

**Fig. 4:**
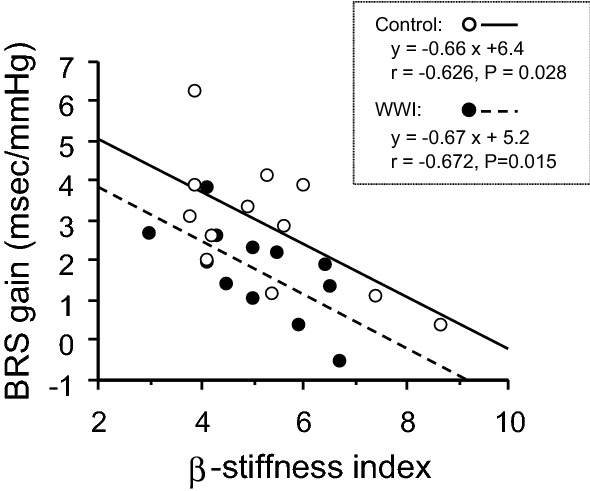
Relationships between carotid arterial β-stiffness index and cardiac baroreflex sensitivity (BRS) gain before (control) and after warm water immersion (WWI)

As shown in Fig. 4, BRS gain correlated with β-stiffness index both the control (*r* = − 0.626, *P* = 0.028) and WWI (*r* = − 0.672, *P* = 0.015) conditions. ANCOVA revealed that the slopes of linear regression line between β-stiffness index and BRS gain remained unchanged after WWI (interaction: *P* = 0.350).

## Discussion

Main results of this study are as follows: first, cardiac BRS gain was blunted after the 5-min WWI. Second, BRS gain correlated with carotid arterial β-stiffness index before (*r* = − 0.626, *P* = 0.028) and after WWI (*r* = − 0.672, *P* = 0.015), and the slopes of linear regression line between β-stiffness index and BRS gain remained similar after WWI. These results indicate that a short-term WWI acutely deteriorates cardiac BRS. Individuals with stiffer arteries are relatively more susceptible to WWI because of their poor baseline BRS, which might be one of the causes of bathing-related falling in elderly persons as well as frailty.

Bath-related accidents (e.g., dizziness and disturbance of consciousness) have been reported frequently in Japan that has a traditional custom to daily warm water immersion (WWI) in the bathtub at the bathing. It is well-known empirically that immediately after WWI feeling dizzy on standing up frequently occurs presumably due to a substantial drop of blood pressure. Because of hydraulic pressure, impacts of aortic, carotid, and cardiopulmonary baroreceptor unloading might be different between supine and standing postures, and thus, response of cardiac BRS to orthostatic challenge with heat stressor would depend on postural setting. Thus, we evaluated cardiac BRS by use of the more practical setting (e.g., stand-up motion after WWI in the bathtub).

The maintenance of BP by baroreflex is critical for preserving cerebral perfusion and orthostatic intolerance. Thus, several studies have been performed to investigate the effect of prolonged passive heat stress accompanied by core temperature elevation on baroreflex control of BP. For instance, whole body heating via water-perfused suits did not alter carotid-cardiac baroreflex function evaluated by the maximal gain between arterial pressure and HR [7, 27, 28], whereas it is decreased when it is evaluated by the relationship between arterial pressure and R–R interval (RRI) [7]. Transfer function analysis reveals that passive heat stress reduces dynamic regulation of cardiac BRS gain [8]. Inconsistent results of baroreflex control of HR might be due to the difference method to evaluate BRS [7, 8]. At least, BRS evaluated using RRI seems to be more sensitive than that based on HR, especially when HR is increased [7]. Given that vagal and sympathetic activity directly influence the slope of pacemaker potential, evaluation using RRI appears to be more reasonable to analyze the stress-associated autonomic response. Accordingly, in the present study, we evaluated cardiac BRS by the relationship between systolic BP and RRI responses.

Prolonged heat exposure with core temperature elevation and hypohydration decreases cardiac baroreflex control of HR [7, 8]. Importantly, the present study indicated that a short-term warm water bathing (e.g., 5 min) without core temperature change deteriorates cardiac BRS. Our results seem to be practically important despite the similarity to previous studies and might extend our physiological understanding of BRS.

High arterial stiffness is associated with lower BRS [18] since the aortic and carotid baroreceptors are stretch receptors. Consistent to the previous study [18], we found that cardiac BRS was significantly and inversely correlated with carotid arterial β-stiffness index before the WWI. Interestingly, after the WWI carotid arterial β-stiffness index remained unchanged, whereas cardiac BRS was significantly blunted. Furthermore, correlation between BRS gain and β-stiffness index was still significant after the WWI with remaining the slopes of linear regression line. Collectively, we might speculate that heat stress did not affect peripheral arc but neural pathway of baroreflex. In any case, individuals with high arterial stiffness (i.e., older populations) may have higher risk for orthostatic intolerance and syncope after the bathing.

In this study, MAP reduced approximately 30 mmHg at the single sit-stand motion from the floor of the bathtub irrespective of conditions. Such instant hypotensive responses were relatively greater than previous studies which used other techniques (i.e., neck chamber, lower body negative pressure, thigh cuff release) [7, 8, 14, 28]. We would like to emphasize that cardiac BRS was blunted by WWI under the present experimental setting; however, no subjects exhibited symptom of pre-syncope on this study. Generally, autoregulation, cardiac BRS, and arterial BRS regulate cerebral perfusion and systemic blood pressure to steady-state against the short-term (i.e., seconds to minutes) [10, 30]. Thus, the other mechanisms including arterial and cardiopulmonary BRS [11] might compensate the deteriorated cardiac BRS.

### Experimental considerations

First, because of the experimental setting (e.g., water immersion in the bathtub), we could not determine sensitivity of aortic, carotid, cardiopulmonary baroreceptors, separately. However, it looks more practical and real-world setting. Second, only men participated in this study. It remains unclear whether WWI similarly affects cardiac BRS in women. Women have lower orthostatic tolerance than men [6, 9, 26], and more commonly faint when exposed to quiescent standing in warm environments [1]. Further studies are warranted among women for generalizability. Third, we posed the potential contribution of hydraulic pressure on the lower body to hemodynamics during hot bathing. However, we failed to answer the question posed because we did not analyze stand-up stress under the presence or absence of hydraulic pressure. Comparison of hemodynamic change among an intact stand-up challenge, after 30 °C (nearly skin temperature) and 42 °C water immersion may allow answering this question.

Heat therapy using hot baths and saunas has been utilized with reports of improved quality of life and overall improved well-being in patients with type II diabetes mellitus [3]. It seems to be a promising intervention to reduce incidence of cardiovascular disease-related mortality, sudden cardiac death and all-cause mortality [12]. On the other hand, a recent prospective survey of the emergency events related to bathing reveals that consciousness disturbance and exhaustion are most frequent symptoms and which are presumably attributed to hyperthermia and dehydration [23]. Regarding this issue, we empathize that despite short-term (e.g., 5 min), cardiac BRS was lowered by warm water bathing which did not elicit remarkable core temperature elevation. Elevation of core temperature and hypohydration are believed to impair orthostatic tolerance [5, 13, 21]. For example, 3% body mass deficit and ~ 0.7 °C concomitant elevation of core temperature had been shown to exacerbate standing-related reductions in the middle cerebral artery blood flow velocity and impair orthostatic tolerance [5]. From the standpoint of safety, further study to determine the impact of prolonged warm water bathing is needed.

## Conclusion

Before and immediately after the 5-min warm water immersion, we compared RRI responses to abrupt drop of SBP induced by the single sit-stand movement in men. The present data indicate that even a short-term warm water immersion deteriorates cardiac BRS and that individuals with stiffer arteries are relatively more susceptible to WWI because of their poor baseline BRS. This might be one of the causes of bathing-related falling in elderly persons as well as frailty. Further studies to determine the impact of prolonged bathing as well as sex difference are needed.

## Data Availability

The datasets used and/or analyzed during the current study are available from corresponding author on reasonable request.

## Abbreviations

BP: Blood pressure
BRS: Baroreflex sensitivity
HR: Heart rate
MAP: Mean arterial pressure
RRI: R–R interval
SV: Stroke volume
WWI: Warm water immersion
